# Skillful and strategic navigation in soccer – a motor-cognitive dual-task approach for the evaluation of a dribbling task under different cognitive load conditions

**DOI:** 10.3389/fpsyg.2024.1356892

**Published:** 2024-06-11

**Authors:** Thomas J. Klotzbier, Nadja Schott

**Affiliations:** Institute of Sport and Movement Science, Department of Sport Psychology and Human Movement Performance, University of Stuttgart, Stuttgart, Germany

**Keywords:** soccer-dribbling, navigation, spatial abilities, motor-cognitive interference, assessment

## Abstract

Soccer is a competitive sport that relies on distinct motor skills and cognitive processes. However, cognitive aspects are often overlooked, with a focus mainly on motor skills. Limited research has explored screening tests within motor-cognitive navigation dual-task (DT) paradigms. This study aims to validate a sensitive approach for assessing soccer-specific dribbling by evaluating the Trail-Dribbling Test (TDT) as a method to differentiate high-performance (HP) from low-performance (LP) players. Two hundred and seventy-five participants (41 females) aged between 12 and 34 completed the Trail-Making Test (TMT), the Trail-Walking Test (TWT), and the soccer-specific TDT under three levels of cognitive load. Results indicated shorter TDT durations for HP compared to LP players, with increased cognitive load accentuating differences (TDT-M: *p* = 0.044, *d* = 0.260; TDT-A: *p* < 0.001, *d* = 0.449; TDT-B: *p* < 0.001, *d* = 0.653). The TDT effectively discriminated between HP and LP players in the 14–15 (AUC = 0.712–0.820) and 16–17 age groups (AUC = 0.634–0.839). In conclusion, the ecologically valid TDT demonstrates the potential for quantifying soccer-specific dribbling, offering insights into motor and cognitive aspects of dribbling performance, especially among soccer players aged 14–17.

## 1 Introduction

Soccer is defined by various specific features and characteristics, such as the specificity and volume of practice, as well as the constraints on performers, including psychological factors, technical and tactical skills, and anthropometric and physiological factors. Additionally, environmental constraints and socio-cultural influences play a significant role (Sarmento et al., 2018). Psychologists and sports scientists employ a wide range of diagnostic tests, ranging from simple to highly complex, to assess different aspects of these cognitive and motor skills. A detailed analysis of the essential characteristics of soccer supports this classification. Soccer is a complex and highly dynamic sport with constantly changing game situations, placing it in the ‘Open Skill Games’ category on the continuum between ‘open’ and ‘closed’ skills (Carling et al., 2007; Nuri et al., 2013). Although certain situations, such as free kicks, corner kicks, kick-offs, and penalty kicks, involve “closed” skills, the majority of skills are performed in complex and changing scenarios. Like most sports games, soccer requires the simultaneous execution of both motor and cognitive skills (Campos, 1993; Casanova et al., 2009). Players must absorb and process relevant information to make effective decisions and plans of action based on their abilities while also possessing the ability to anticipate the game (Williams, 2000). Perceptual tasks, such as tracking the ball and the movements of teammates and opponents, assessing player positions on the field, understanding tactical alignment, interpreting the coach’s instructions, and considering the current game situation, must be integrated into their decision-making processes and action plans (Sakamoto et al., 2018). However, when it comes to assessing motor skills, these aspects have rarely been considered.

### 1.1 Cognitive test procedures

Ali (2011) provides an interesting review of cognitive and motor test procedures in soccer, identifying strengths and weaknesses and offering methodological testing recommendations. Two approaches can be identified regarding the cognitive tests discussed in the review. The first approach, the “Cognitive Component Skill Approach,” focuses on general cognitive skills that differentiate experts from novices. This approach often utilizes paper-pencil methods or computer-based reaction time experiments. While these methods offer high internal validity, they may be limited in ecological validity and their ability to quantify the intricate cognitive processes during a soccer game. Consequently, sports training is viewed as cognitive training, leading to structural and functional adaptations that enhance cognitive performance (referred to as the “Cardiovascular Fitness Hypothesis”; Aberg et al., 2009; see also Audiffren and André, 2019). However, Beavan et al. (2020a,b) have questioned this approach, particularly the relationship between sport-specific experience and cognitive skills. They raise concerns about incorporating cognitive skills in the process of talent identification. An alternative approach is the “Expert Performance Approach,” which assesses athletes in ecologically valid contexts using tasks that are representative of their specific domain. Mann et al. (2007) conducted a meta-analysis demonstrating that experts recognize domain-specific cues faster and process them more effectively. Experts also exhibit different strategies in visual search tasks than novices, showing fewer fixations (saccade jumps) of longer duration. These tests often involve video-based experiments or simulations of game situations that may not necessarily be observed from a first-person perspective. However, it is important to consider that decision-making and anticipation processes may differ fundamentally in real-game situations, raising questions about the transferability of this methodological approach (Roca et al., 2011). Furthermore, determining correct answers in these tests could rely on the subjective decisions of trainers, researchers, or test administrators. A possible transfer effect as well as the dependence on subjective decisions about correct answers necessitate further research efforts.

Musculus et al. (2022) and Knöbel and Lautenbach (2023) aimed to combine the strengths of both approaches to develop and validate cognitive tasks for measuring inhibition, cognitive flexibility, and working memory in a soccer-specific context. The tasks were paired with a soccer-specific motor response (i.e., pass). The authors suggest that utilizing this approach allows for an effective assessment of core executive functions. They propose that these tasks could serve as a reliable cognitive diagnostic tool for soccer clubs.

### 1.2 Assessing relevant soccer-specific motor skills for talent diagnostics

Procedures for assessing motor skills in soccer often focus on isolating specific aspects, such as passing or shooting, typically in static or otherwise controlled situations. However, according to Ali (2011), the cognitive component is a fundamental part of a skill, encompassing decision-making and information processing. Unfortunately, many tests tend to neglect this cognitive component, which raises doubts about their ecological validity, as per Ali’s (2011) definition of skills.

When conducting talent research, it is crucial to consider not only the quality of test procedures used to assess cognitive and motor skills but also which characteristics are considered relevant (Williams and Reilly, 2000; Kannekens et al., 2011). Talent development is a multifactorial process that depends on a variety of individual factors and environmental factors (Murr et al., 2018). This raises the question of which criteria are crucial for identifying talents and how these requirements should be specified. It is important to note that this requirement profile is position-specific. For example, the ability to score goals may be highly relevant for a striker but considered less critical for a defender. There are also numerous examples of exceptional soccer players, such as Lionel Messi or Andres Iniesta, who excel in the sport despite not possessing exceptional heading skills. Therefore, this study introduces a test procedure to quantify dribbling as a vital component in soccer.

### 1.3 Integration of motor and cognitive components in navigation (dual) tasks

Dual-task (DT) paradigms provide a valuable opportunity to integrate the cognitive component and combine motor and cognitive tasks (see Box 1). From a methodological perspective, cognitive-motor interference has seldom been studied in physical environments with high variability of spatial movements (Smith and Chamberlin, 1992; Beilock et al., 2002). Previous studies have mainly focused on ecologically less valid laboratory situations, such as straight-ahead walking on a treadmill or at ground level, which reduces the demands on physical navigation (Moreira et al., 2021). Treadmills are valuable tools for analyzing gait kinematics during continuous walking (e.g., Padulo et al., 2014). However, they only allow for straight-line walking, which is a mechanical and continuous motor act that does not require the same level of attention to the environment as walking in everyday situations or during sports. Visual input is crucial in the interplay between the body and the environment (Imai et al., 2001). However, comprehensive situational awareness, spatial orientation, and the ability to move freely in three-dimensional space are also essential for successful action in ball sports (Woods et al., 2020; Bartseva et al., 2024).

In sports, players often navigate from point A to point B, constantly directed toward a physical object (e.g., the ball, a teammate) or a place (e.g., our own or the opponent’s goal) to perform a specific action (e.g., pass, shot on goal). The term “navigation” is defined as coordinated and goal-oriented movement through space, which is made up of two components: Wayfinding and Locomotion (Montello, 2005; Wiener et al., 2009). Wayfinding is the planning and problem-solving part of navigation in which decisions are made. This involves planning and deciding on a series of actions (e.g., “pass to teammate” + “run deception” + “run into the free gap”) based on available information and existing knowledge about the space. To do this, one refers to internal (existing knowledge about the space [e.g., the soccer field] in your head) or external knowledge (e.g., specific moves). During “route planning,” individuals tend to identify potential running paths that match their goals and then use various implicit and explicit strategies to quickly reduce the options and settle on a route (e.g., a direct running path to the goal). During the execution of the plan – the locomotion – it is constantly reviewed based on the newly received information from the environment and, if necessary, adjusted (e.g., when a free gap closes) (Montello, 2005; Yang et al., 2018). The locomotion is the pure movement through space in the direction specified by the plan. During this movement, the immediate surroundings are primarily perceived to avoid possible obstacles. Although these two components can be described separately, they rarely occur independently and can, therefore, be described as a dual task (Brunyé et al., 2018). Separating locomotion and wayfinding only occurs when walking aimlessly or planning a game move that is not executed (Montello and Sas, 2006). Thus, successful navigation involves efficiently reaching a specific destination without causing physical harm. This requires awareness of one’s location in relation to the destination and other places or objects while in motion.

In the context of soccer-specific navigation, two intriguing studies have been conducted that address distinct research questions while incorporating both motor and cognitive demands in a DT dribbling test. Smith and Chamberlin (1992) asked female soccer players of varying performance levels to complete three different conditions. Participants were instructed to complete a slalom course emphasizing running agility in the first condition. The goal was to finish as quickly as possible without dribbling a soccer ball. In the second condition, participants had to dribble through the course as rapidly as possible. In the third condition, participants had to identify various symbols displayed on a screen while simultaneously performing the dribbling task. Comparing the three groups, experts demonstrated significantly less interference than the other groups. The differences between the expert groups became more pronounced when the cognitive component was performed in parallel with the motor task. In the second study by Beilock et al. (2002), dribbling was explored as a motor skill within a DT paradigm and involved two groups: experts and novices. Both groups were tasked with dribbling the ball through a slalom course using either their dominant or non-dominant foot while simultaneously performing additional cognitive tasks. These tasks included identifying auditory stimuli or directing attention solely to dribbling (skill-focused). The results of the study indicated that completing the DTs took longer for both groups, with one notable exception: the expert group exhibited faster performance when dribbling with their dominant leg. Interestingly, in the skill-focused attention condition, both experienced and novice participants dribbled at a more similar speed. The contrast between the two groups became more evident during the DT condition, especially when dribbling with the dominant leg. This highlights that experienced performers demonstrated a significant speed advantage over novices in the DT condition. However, in the skill-focused condition, this advantage was considerably diminished when dribbling with the dominant leg. In summary, the study showed that experienced soccer players performed notably faster in the DT condition compared to the skill-focused condition, while novices displayed a tendency toward the opposite pattern, dribbling faster in the skill-focused condition than in the DT condition.

These two studies are intriguing because they separate cognitive (wayfinding) and motor tasks (dribbling), allowing them to be performed independently. This creates the possibility of prioritizing one task over the other, potentially neglecting one of the two. In addition, both studies (Smith and Chamberlin, 1992; Beilock et al., 2002) used non-soccer-related cognitive tasks without direct reference to the dribbling task. In terms of the navigation required in soccer, a dual task that establishes a greater connection to soccer-specific path planning and execution would, therefore, be advantageous. An elegant way to test visual–spatial abilities with different cognitive loads in a soccer-related navigation task is provided by the adaptation of the Trail-Walking Test (TWT; Schott, 2015). In this study, we employ both non-soccer-specific tasks (TWT) and a soccer-specific adaptation of the TWT (Trail-Dribbling Test; TDT).

**BOX 1 A critical view and alternative perspectives on constructing DT paradigms.**

Multitasking is a broad concept that can be measured in various ways (Kiesel et al., 2022). It involves performing multiple tasks, each associated with a separate task set, within a limited period of time, resulting in a temporal overlap of cognitive processes (Koch et al., 2018). A task can be defined as an abstract description of a future state or a desired goal. It can be instructed, where task sequences are predetermined, or self-organized, where they are functionally interdependent (e.g., wayfinding) or independent (e.g., continuous motor task and counting backward in steps of 3; Künzell et al., 2018; Strayer et al., 2022). Furthermore, a task set refers to a representation of the cognitive and motor requirements necessary to perform a task (Koch et al., 2018).

Temporal overlaps of cognitive processes can occur during the execution of multiple tasks, such as in task switching (i.e., sequential multitasking) and dual-tasking (i.e., simultaneous multitasking). Sequential multitasking involves performing one task for an extended period before switching to another task. This can range from several minutes, such as walking while having a conversation, to even hours, such as cooking and reading a book (Salvucci and Taatgen, 2011). However, in concurrent multitasking, tasks are performed simultaneously or with frequent switches between them in short periods of less than 1 min. This can result in lower performance in one or both tasks (Koch et al., 2018).

McIsaac et al. (2015) propose a standardized classification system for tasks in motor-cognitive dual-task paradigms. They define a dual-task as two separate tasks with different goals that are functionally independent of each other (see also Strayer et al., 2022). They have different goals (e.g., walking vs. talking) with different stimuli (the environment vs. the content of the conversation) and responses (e.g., avoiding an obstacle vs. speaking and listening). It is crucial to note that the performance of each task can be evaluated separately. We critically question McIsaac’s taxonomy and discuss the nature and construction of DTs without prescribing a single standard for their design.

Our approach is based on the idea that it is not possible to completely neglect one task when people are asked to perform two tasks simultaneously, such as walking and counting backward. This is especially important considering the importance that participants attribute to the cognitive task. This discovery presents a challenge for designing DT experiments and emphasizes the constraints of previous research methods, especially regarding common daily situations that frequently involve interdependent tasks. For instance, when driving through an unfamiliar area, it is important to be able to follow navigation instructions simultaneously. This is a situation in which cognitive processes must perform multiple tasks in parallel or at least partially in parallel (Koch et al., 2018). Unlike functionally independent tasks, where one task can be neglected, the Trail Dribbling Test used here emphasizes the need to pay attention to both tasks.

For instance, a player could dribble to the letter B while simultaneously performing a visual search for the number 3. While dribbling, the cognitive task can be neglected, and the motor component of dribbling to be impaired while moving slowly and searching visually. However, it is important to integrate both tasks into a coherent action plan that allows the player to continue searching for the next number while dribbling. Experienced players are more likely to achieve this, particularly if dribbling is automatic and there are enough cognitive resources for both tasks. Additionally, motor and cognitive tasks can be scored separately, such as dribbling through the parkours versus reciting or showing the Trail Making Test conditions A and B with a laser pointer. By calculating the dual-task costs (DTC), it is possible to examine the distribution of resources between the tasks.

Overall, we believe that the definition of what constitutes DT remains subject to interpretation, lacking a universally accepted standard for their design.

The overall goals of this study were (1) to investigate the feasibility of differentiating performance groups (novices vs. experts) using two different navigational DTs that vary in their degree of specificity to soccer, and (2) to determine the specific age ranges in which differentiation between the performance groups is possible, and (3) to evaluate the sensitivity and specificity of this differentiation. It was hypothesized that varying levels of interference would occur depending on the specificity of the tasks and that a soccer-specific skill would be more likely to differentiate between younger high-performance (HP-SP) and low-performance soccer players (LP-SP). Specifically, it is assumed that as the cognitive load of the navigation (dual) tasks increases, the differences between HP-SP and LP-SP would become more pronounced, particularly in soccer-specific tasks, compared to situations with low cognitive load and soccer-unspecific tasks.

## 2 Methods

### 2.1 Sample size estimation

Power analysis [using G*Power3; a statistical power analysis program (Faul et al., 2007)] was conducted to estimate the necessary sample size. In our ANCOVA analysis with repeated measures with one covariate, we aimed for a 95% power, an effect size of *f* = 0.25, and a significance level of *p* = 0.05 to identify fixed, main, and interaction effects. The calculated sample size needed was 251 participants. Our sample size of 275 exceeds the necessary number, ensuring our desired statistical power and confidence level.

A power analysis (Superpower; Caldwell and Lakens, 2019) was conducted on a 2(group) x 2(domain) x 3(condition) ANOVA with repeated measures design with 20 participants per cell (12 cells in our design). Assuming a high effect size of *f* = 0.4, a standard deviation of 1.0, a correlation of 0.5, and an alpha of 0.05, a power of 100% was found for the main effect of group, a power of 100% for the main effect of condition, a power of 81.1% for the main effect of domain, a power of 6.3% for the interaction effect of group x condition, 5.6% for the interaction effect of group x domain, 5.5% for condition x domain, and 5.7% for the three-way interaction of group x condition x domain. Due to the limited power for detecting interaction effects within our analyses, we can only provide reliable statements regarding the main effects, which aligns with the recommendation made by Brysbaert (2019).

### 2.2 Participants

The players were recruited from amateur and professional soccer clubs across Germany, including players from the youth development centers of top-class clubs such as VfB Stuttgart or FC Schalke 04. The participants were primarily recruited from clubs in the Stuttgart region. All potential participants from the mentioned clubs who volunteered to participate in the study were included. Individuals with motor or cognitive impairments were excluded. Swann et al. (2015) developed a classification system based on the athlete’s highest level of performance, success, experience, and competitiveness (national and global). This system categorizes players into four levels of elite competitive athletes. Key variables of their definition are the highest standard of performance as well as success and experience at the athlete’s highest level. Since this is aggregated data from various surveys, not all participants were initially queried on these essential variables for Swann’s categorization. However, we were able to determine the competition level for all participants. Depending on the competition level, participants were assigned to either the high-performance (HP) or low-performance (LP) group: Participants playing in an active adult team at the “Landesliga” (1st to 6th division) level or above were assigned to the HP group. In contrast, those playing below the “Bezirksliga” (8th + division) level were assigned to the LP group. In youth teams, participants playing in the “Verbandsliga” were assigned to the HP group, while those below the “Bezirksliga” level were assigned to the LP group. Athletes who had previously played at a high level but were not actively playing soccer were excluded.

### 2.3 Instruments

#### 2.3.1 Cognitive control

The Trail-Making Test (TMT; Reitan, 1958) assesses cognitive processing speed, executive functions, and attentional components (Salthouse, 2011). The test comprises of two parts. In part A, the test subjects are to connect circles numbered from 1 to 25 in ascending order and as quickly as possible. In part B, the test subjects are to connect the numbers 1 to 13 and the letters from A to L alternately in ascending order and at maximum speed. Furthermore, a motor speed-tracking task measures the participant’s fine-motor performance (Schott et al., 2016). The task records the time taken and the number of errors made. Any shifting and sequential errors are immediately corrected by the examiner, who instructs the participant to return to the last correct circle. Therefore, errors are factored into the required times as correcting errors takes additional time (Schott et al., 2016; Klotzbier et al., 2020). Each trial and sequence is carried out until the last cone is reached. The same approach concerning shifting and sequential errors is used for the two subsequent DTs.

#### 2.3.2 Non-soccer-specific and soccer-specific dual-tasks

**Figure 1:**
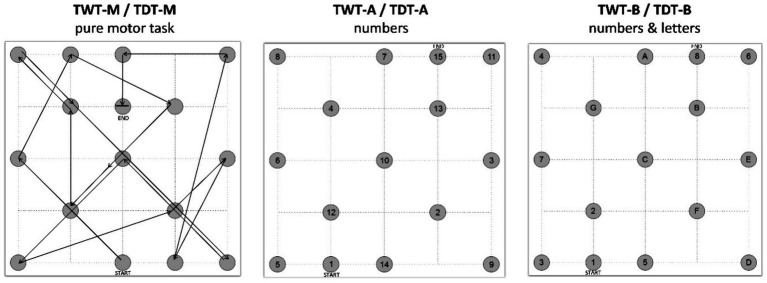
Conditions of the Trail-Walking Test and the Trail-Dribbling Test (Schott, 2015). TWT-M, Trail-Walking Test – pure motor task; TDT-M, Trail-Dribbling Test – pure motor task; TWT-A, Trail-Walking Test – numbers; TDT-A, Trail-Dribbling Test – numbers; TWT-B, Trail-Walking Test – numbers & letters; TDT-B, Trail-Dribbling Test – numbers & letters.

The Trail-Walking Test (TWT, Schott, 2015) is conducted on a 4×4 meter playing field consisting of 15 cones labeled with numbers or numbers and letters depending on the condition. In the motor condition (TWT-M), the objective is to navigate a designated path as quickly as possible. In this condition, the cones do not have any numbers or letters; only the path on the playing field is marked with chalk. In the second condition (TWT-A), participants must run to the cones in ascending numerical order (1-2-3-…-15). In the third and final condition (TWT-B), the task is to run to the cones in alternating ascending order of numbers and letters (1-A-2-B-…-8). In addition, there was another implementation format for an alternative calculation of the cognitive costs (signaling condition). Here, the participants (subsample *n* = 165) stood in the middle of the field and had to use a laser pointer to (1) trace the purely motor path, (2) connect the numbers, and (3) the numbers and book strokes as quickly as possible. The objective is to complete the course as fast as possible without making any errors. The same conditions are applied in the Trail-Dribbling Test (TDT), except participants must dribble a soccer ball through the course. All conditions’ lengths are identical (41 meters) for accurate comparisons. Stopwatch measurements are used to record the times, rounded to 0.01 s. The positions of the cones placed in the field, as shown in the run schedule in Figure 1, remained the same for each condition and each trial. For the purely motor condition without soccer-specific skill (TWT-M), there was one practice session to become familiar with the run schedule. Each condition, including both the non-soccer-specific task and the soccer-specific task, was conducted three times.

### 2.4 Procedure

The data was collected during the teams’ scheduled training sessions. Following the demographic data questionnaire, all tests and conditions (TMT, TWT, TDT) were randomized to avoid sequence effects. Each condition was conducted three times, and the mean within each condition was calculated. Data from individual trials are not accessible due to data pooling; however, we do have data from a total of 42 participants (both LP and HP) across all three trials to capture the learning effect in the TWT and TDT. A 3-min break was taken between each run to eliminate possible fatigue effects. Informed written consent was obtained from the clubs/organizations and parents/guardians before the testing. Additionally, participants provided consent and were informed of their right to withdraw from the study at any time. The informed consent was given willingly and without any coercion or bribery. All procedures adhered to the principles outlined in the Declaration of Helsinki (World Medical Association, 2013), including ethical standards, legal requirements, and international norms.

### 2.5 Data analysis

SPSS v.27 (SPSS, Chicago, IL) was used for the statistical analysis. Initially, we assessed missing data points, evaluated the normality of distributions (using Kolmogorov–Smirnov tests), and checked for extreme values in the dependent variables. A significance level of 0.05 was utilized for all statistical tests, following the guidelines of Tabachnick et al. (2013). We used Eta-squared (ɳ^2^_p_) and calculated Cohen’s d as effect size measures.

#### 2.5.1 Sample characteristics

T-tests were employed for continuous variables to evaluate potential differences in baseline characteristics between groups, such as age, BMI, and years of regular training. A chi-square test was used for categorical demographic variables such as sex.

#### 2.5.2 Velocities for the TMT

We calculated the velocities for the TMT to account for the different lengths in the conditions of the TMT (TMT-M: 185.4 cm; TMT-A: 185.4 cm and TMT-B: 243.8 cm; Gaudino et al., 1995). The absolute durations were used for the TWT conditions (TWT-M, TWT-A, and TWT-B) and the TDT conditions (TDT-M, TDT-A, and TDT-B) since the lengths are the same across conditions.

#### 2.5.3 Dual-task costs (DTC)

The performance in each task under DT conditions is compared to the performance in the respective single-task (ST) conditions. Negative signs have been inserted to indicate poorer performance in the DT conditions compared to ST conditions. Therefore, negative DTC values represent a deterioration in performance, while positive DTC values indicate a relative improvement in performance under DT conditions (Plummer and Eskes, 2015, p. 3). Motor and cognitive DTC were computed for both the number condition and the number and letter condition in both the soccer-specific (TDT) and non-soccer-specific (TWT) tasks.


$$DTC=\frac{-\left(DT\;\mathrm{performance}-ST\;\mathrm{performance}\right)}{ST\;\mathrm{performance}}*100$$

To calculate the cognitive ST performance, the purely motor conditions (TWT-M or TDT-M) were subtracted from the DT conditions. This was used to obtain the time for the ST cognitive process. In addition, an alternative method for calculating cognitive DTC was used for a subsample of 165 individuals. The time required for the signaling task served as a measure of cognitive performance under ST conditions. Subsequently, the calculation of DTC was also carried out according to the formula specified above.

#### 2.5.4 Analysis of variance

A 2 (group: LP-SP vs. HP-SP) x 3 (condition: only motor, numbers, numbers and letters) ANCOVA with repeated measurement for the calculated times in the TWT and TDT and age as covariate was performed to test the effect of the three different cognitive conditions.

For the evaluation of DTCs, a 2 (group: LP-SP vs. HP-SP) x 2 (condition: only motor, numbers, numbers and letters) x 2 (domain: motor vs. cognitive) ANCOVA with repeated measurement and age as a covariate was performed. Group differences within the conditions (e.g., TWT-M) were investigated using t-tests for independent samples (Bonferroni correction). In addition to the significance value (*p* < 0.05, *significant; *p* < 0.01; strong significant; *p* < 0.001, highly significant), the effect sizes for all ANCOVAs are given using Partial Eta Squared (ɳ^2^_p_).

#### 2.5.5 Receiver operating characteristic (ROC) analyses

Sensitivity, specificity, and the area under the curve (AUC) were considered as quality measures to evaluate the diagnostic capability of the TDT. Participants were categorized into distinct age categories: (see Table 1) to investigate the diagnostic accuracy across different age groups. The Youden index was utilized to determine the optimal threshold for distinguishing between LP and HP individuals within each age group. This index, calculated as Youden index = (sensitivity + (specificity-1)), can range from −1 to 1 (Hilden and Glasziou, 1996).

**Table 1:** Participant characteristics in LP-SP and HP-SP across age groups for evaluating receiver operating characteristics.

	LP-SP 12–13	HP-SP 12–13	LP-SP 14–15	HP-SP 14–15	LP-SP 16–17	HP-SP 16–17	LP-SP 18–24	HP-SP 18–24	LP-SP 25–34	HP-SP 25–34	Statistical analysis (*n* = 275)
	(*n* = 26)	(*n* = 55)	(*n* = 27)	(*n* = 15)	(*n* = 9)	(*n* = 31)	(*n* = 56)	(*n* = 31)	(*n* = 15)	(*n* = 10)	
Age (years)	12.4 (0.50)	12.5 (0.50)	14.2 (0.42)	14.3 (0.49)	16.3 (0.50)	16.4 (0.50)	21.5 (2.04)	21.0 (1.73)	28.2 (2.59)	28.5 (2.42)	*F*(9,265) = 391***, ɳ^2^_p_ = 0.930
	*t*(106) = −0.395^ns^		*t*(40) = −0.772^ns^		*t*(38) = −0.453^ns^		*t*(85) = 1.11^ns^		*t*(23) = −0.291^ns^		
Sex	26 ♂, 0 ♀	55 ♂, 0 ♀	14 ♂, 13 ♀	14 ♂, 1 ♀	4 ♂, 5 ♀	16 ♂, 15 ♀	53 ♂, 30 ♀	27 ♂, 4 ♀	15 ♂, 0 ♀	10 ♂, 0 ♀	*CHI^2^*(1) = 135.5***
	*N/A*		*CHI^2^*(1) = 7.47**		*CHI^2^*(1) = 0.143^ns^		*CHI^2^*(1) = 1.54^ns^		*N/A*		
BMI (kg/m^2^)	17.4 (1.81)	18.1 (1.87)	19.4 (2.83)	19.3 (2.20)	20.4 (2.09)	21.0 (1.39)	22.9 (1.90)	22.7 (1.59)	25.1 (1.58)	24.4 (1.95)	*F*(9,242) = 38.6***, ɳ^2^_p_ = 0.590
	*t*(106) = −0.949^ns^		*t*(39) = 0.068^ns^		*t*(38) = −1.08^ns^		*t*(84) = 0.350^ns^		*t*(23) = 0.966^ns^		
Age of regular training? (years)	*N/A*	5.56 (1.56)	9.39 (3.24)	5.47 (1.68)	10.7 (3.71)	6.20 (2.28)	7.96 (2.79)	6.38 (2.37)	8.36 (4.60)	5.56 (1.87)	*F*(8,200) = 7.76***, ɳ^2^_p_ = 0.273
	*N/A*		*t*(38.7) = 5.09***, *d* = 1.64		*t*(9.88) = 3.43*, *d* = 1.11		*t*(81) = 2.59*, *d* = 0.576		*t*(21) = 1.73^T^, *d* = 0.755		
Amount of training in soccer (min/week);	*N/A*	360 (0.00)	184 (29.7)	354 (23.2)	200 (75.0)	312 (57.8)	212 (106)	316 (110)	198 (51.7)	325 (57.5)	*F*(8,208) = 19.9***, ɳ^2^_p_ = 0.434
	*N/A*		(40) = −19.1***, *d* = −6.04		t(38) = −4.81***, *d* = −1.56		(85) = −4.28***, *d* = −0.928		*t*(23) = −5.78***, *d* = −2.41		
TMT-M (motor task)	*N/A*	*N/A*	9.39 (2.78)	6.55 (0.587)	8.16 (0.988)	6.29 (2.05)	9.19 (2.24)	8.92 (2.37)	10.5 (2.45)	9.89 (2.79)	*F*(7,129) = 7.03***, ɳ^2^_p_ = 0.276
	*N/A*		*t*(29.1) = 4.74***, *d* = 1.76		*t*(38) = 2.64***, *d* = 0.857		*t*(45) = 0.408^ns^		*t*(16) = 0.453^ns^		
TMT-A (numbers)	*N/A*	*N/A*	24.7 (10.1)	21.5 (5.64)	19.3 (5.97)	19.2 (6.97)	20.2 (5.23)	17.1 (5.74)	21.6 (2.72)	17.3 (2.18)	*F*(7,129) = 2.78^T^, ɳ^2^_p_ = 0.131
	*N/A*		*t*(30) = 0.675^ns^		*t*(38) = 0.007^ns^		*t*(45) = 1.99^T^, *d* = 0.593		*t*(16) = 3.62**, *d* = 1.81		
TMT-B (numbers & letters)	*N/A*	*N/A*	52.9 (15.4)	56.2 (15.6)	44.2 (11.1)	47.8 (17.0)	44.9 (12.9)	44.7 (13.4)	37.9 (5.93)	37.1 (13.2)	*F*(7,129) = 2.29*, ɳ^2^_p_ = 0.111
	*N/A*		*t*(30) = −0.434^ns^		*t*(38) = −0.607^ns^		*t*(45) = 0.049^ns^		*t*(16) = 0.180^ns^		

The age ranges are also used to evaluate the TDT as a diagnostic test; N/A, not available (due to organizational constraints, capturing the TMT was not always feasible); HP-SP, high-performance soccer players; LP-SP, low-performance soccer players; NS, not significant; T, tendencially significant; *p* < 0.05, *significant; *p* < 0.01; strongly significant; *p* < 0.001, highly significant; BMI (Body Mass Index), Weight (in kilograms) / (Height (in meters) * Height (in meters)); TMT-M, Trail-Making Test – pure motor task; TMT-A, Trail-Making Test – numbers; TMT-B, Trail-Making Test – numbers & letters; ɳ^2^_p_, Partial Eta Squared; ♂, male; ♀, female.

## 3 Results

### 3.1 Participants

A total of 275 participants were included in the study, comprising 234 males and 41 females. There were no significant differences in sex distribution between the groups, with a higher proportion of males in both groups. Of the participants, 242 identified soccer as their primary sport. The high-performance (HP-SP) group was significantly younger than the low-performance (LP-SP) group. The HP-SP group had a lower BMI than the LP-SP group. The HP-SP group began their regular training significantly earlier than the LP-SP group. Furthermore, the HP-SP group had more training hours in their current sport than the LP-SP group. Only 135 participants completed the Trail-Making Test (TMT) (equally distributed between the performance groups: 69 in HP-SP and 68 in LP-SP). The HP-SP group differed significantly from the LP-SP group in conditions M and A of the TMT, but no differences were observed in the condition with high cognitive load (TMT-B) (see Table 2).

**Table 2:** Participant characteristics of the LP-SP and HP-SP.

	LP-SP (*n* = 133)	HP-SP (*n* = 142)	Statistical analysis
Age (years)	18.7 (5.31)	16.5 (4.75)	*t*(273) = 3.49, *p* = 0.001, *d* = 0.422
Sex	112 male, 21 female	122 male, 20 female	*CHI^2^*(1) = 0.16, *p* = 0.692
BMI (kg/m^2^)	21.8 (3.03)	20.4 (2.75)	*t*(250) = 3.97, *p* < 0.001, *d* = 0.502
How old were you when you started regular training? (years)	8.61 (3.34)	5.95 (2.06)	*t*(169) = 6.91, *p <* 0.001, *d* = 1.063
Amount of training in soccer (min/week); freq. of naming soccer (n)	202 (83.9) *n =* 84	330 (70.9) *n =* 110	*t*(215) = −12.12, *p <* 0.001, *d* = −1.653
TMT-M; motor task (s)	9.31 (2.42)	7.66 (2.60)	*t*(135) = 3.85, *p* < 0.001, *d* = 0.663
TMT-A; numbers (s)	22.0 (7.63)	18.4 (6.11)	*t*(135) = 3.07, *p* = 0.003, *d* = 0.528
TMT-B; numbers& letters (s)	46.9 (13.8)	46.1 (15.8)	*t*(135) = 0.334, *p* = 0.739, *d* = 0.057

Mean values and standard deviations as well as statistical parameters, are given. HP-SP, high-performance soccer players; LP-SP, low-performance soccer players; BMI (Body Mass Index), Weight (in kilograms) / (Height (in meters) * Height (in meters)); TMT-M, Trail-Making Test – pure motor task; TMT-A, Trail-Making Test – numbers; TMT-B, Trail-Making Test – numbers & letters; s, seconds; n, frequency; *p* < 0.05, significant; *p* < 0.01; strongly significant; *p* < 0.001, highly significant; d, Cohens d.

The ROC analyses did not reveal any age differences between the high-performance (HP-SP) and low-performance (LP-SP) groups (see Table 1). However, there were significant differences in sex distribution between the performance groups, with a higher proportion of males than females. It is worth noting that only male participants were tested in the youngest and oldest age groups. Furthermore, it was observed that the HP-SP group had more training hours in soccer than the LP-SP group (*d* = −0.928 – −6.04).

### 3.2 Durations in the non-soccer-specific task (TWT) and the soccer-specific task (TDT)

#### 3.2.1 Trail-walking test

**Figure 2:**
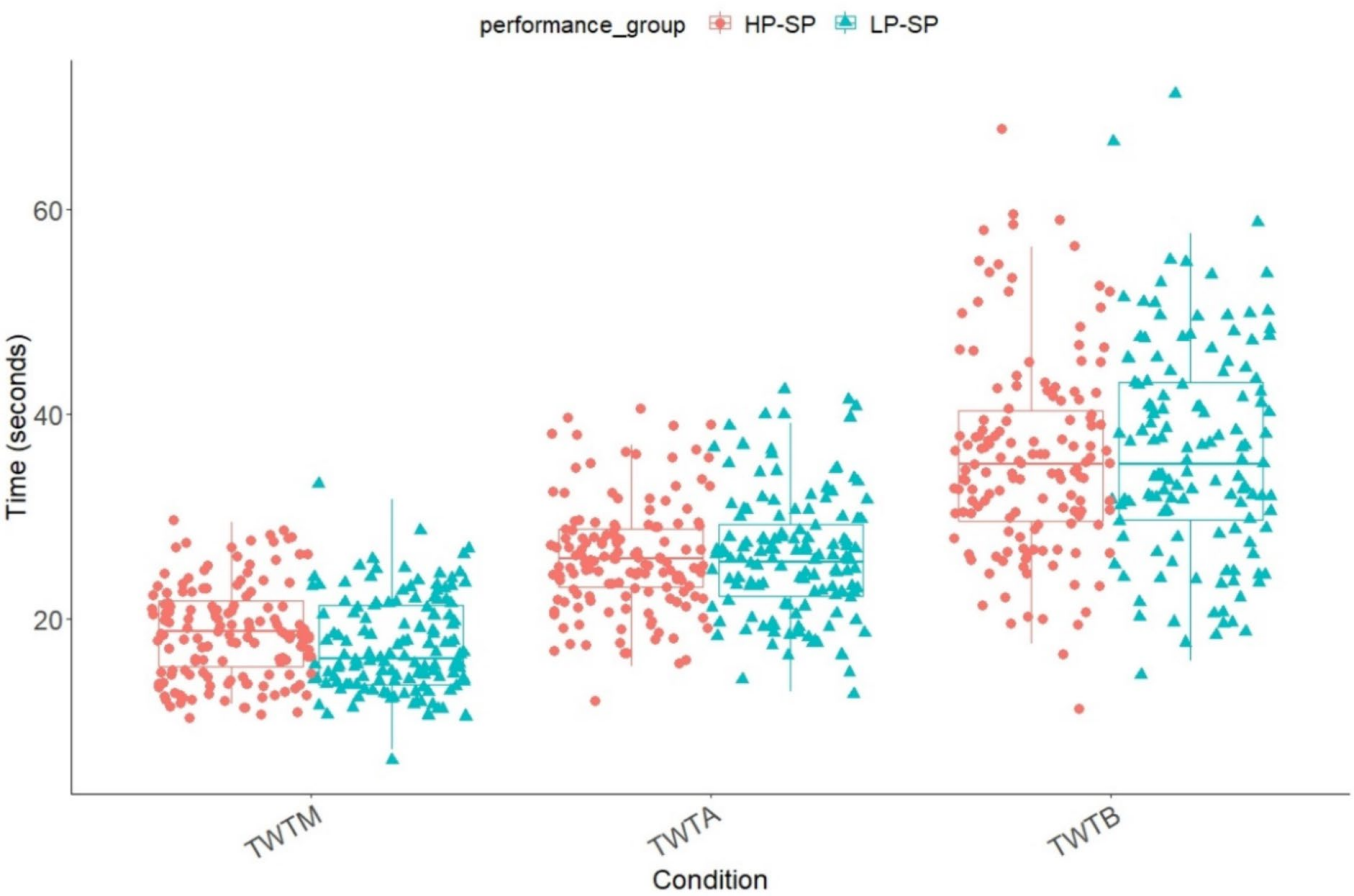
Means and standard deviation of groups and conditions in the TWT (TWT-M, TWT-A & TWT-B) based on the times. TWT-M, Trail-Walking Test – pure motor task; TWT-A, Trail-Walking Test – numbers; TWT-B, Trail-Walking Test – numbers & letters.

The durations in all three conditions of the TWT and all groups were normally distributed (*p* < 0.001). Age correlated (Pearson correlation coefficient) significantly with the durations in all conditions (TWT-M: *r* = −0.692, *p* < 0.001; TWT-A: *r* = −0.159, *p* = 0.009; TWT-B: *r* = −0.206, *p* < 0.001). Sex did not influence the performance in any TWT conditions (TWT-M: *p* = 0.089; TWT-A: *p* = 0.079; TWT-B: *p* = 214). The mean values of the TWT in ST and DT are shown in Figure 2 for both performance groups. Participants show improvement across the three trials and in all three conditions of the TWT (TWT-M: 14.7–14.5 – 14.3; TWT-A: 30.0–25.7 – 23.9; TWT-B: 39.2–33.4 – 31.2).

A 2 (group: HP-SP vs. LP-SP) x 3 (condition: motor, numbers, numbers and letters) ANCOVA with repeated measures of TWT durations and age as covariate showed significant main effects for condition, *F*(1.57, 425) = 50.7, *p* < 0.001, ɳ^2^_p_ = 0.158, and age *F*(1, 270) = 43.1, *p* < 0.001, ɳ^2^_p_ = 0.138. A significant group difference was not observed, *F*(1, 270) = 0.827, *p* = 0.364, ɳ^2^_p_ = 0.003. In addition, ANCOVA led to a significant interaction of condition x age, *F*(1.57, 425) = 10.2, *p* < 0.001, ɳ^2^_p_ = 0.036. The interaction condition x group was not significant, *F*(1.57, 425) = 1.35, *p* = 0.256, ɳ^2^_p_ = 0.005. The post-hoc analysis showed that the durations in TWT-B (*M* = 36.2, *SE* = 0.597) were significantly higher than in TWT-A (*M* = 26.1, *SE* = 0.341) or the motor (TWT-M) condition (*M* = 18.0, *SE* = 0.198) (*p* < 0.001). Also, the *post hoc* analysis showed that for the TWT-M, both groups differ significantly from each other, *t*(273) = −2.72, *p* = 0.007, *d* = −0.328.

#### 3.2.2 Trail-dribbling test

**Figure 3:**
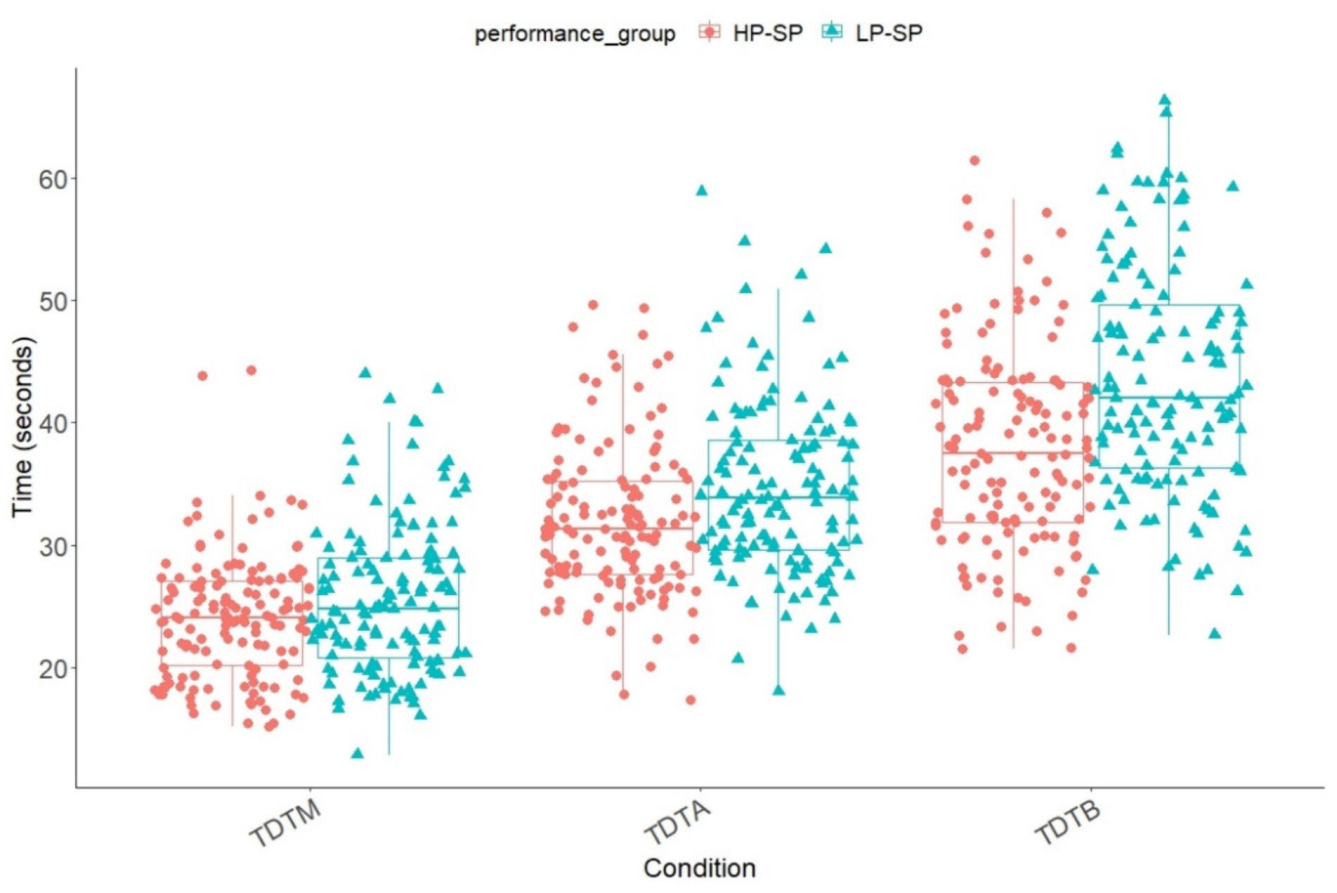
Means and standard deviation of the groups and conditions of the TDT (TDT-M, TDT-A & TDT-B) based on the times. TDT-M, Trail-Dribbling Test – pure motor task; TDT-A, Trail-Dribbling Test – numbers; TDT-B, Trail-Dribbling Test – numbers & letters.

The durations in all three conditions of the TDT and all groups were normally distributed (*p* < 0.001 – *p* = 0.007). The age correlated (Pearson correlation coefficient) significantly with the durations in the TDT-M (*r* = −0.501, *p* < 0.001) but not in the conditions with cognitive load (TDT-A: *r* = −0.040, *p* = 0.254; TDT-B: *r* = −0.004, *p* = 0.473). Sex influenced the performance in the TDT in all conditions (*p* < 0.001), where males produced lower durations in all conditions. The mean values of the TDT ST and DT conditions are shown in Figure 3 for both performance groups. Participants show improvement across the three trials and in all three conditions of the TDT (TDT-M: 19.7–19.5 – 19.0; TDT-A: 34.8–32.8 – 29.7; TDT-B: 44.0–40.3 – 36.9).

A 2 (group: HP-SP vs. HP-SP) x 3 (condition: motor, numbers, numbers, and letters) ANCOVA with repeated measures of TDT durations and age as a covariate showed significant main effects for condition, *F*(1.81, 488) = 14.5, *p* < 0.001, ɳ^2^_p_ = 0.051, group, *F*(1, 269) = 32.1, *p* < 0.001, ɳ^2^_p_ = 0.107, and age *F*(1, 269) = 18.7, *p* < 0.001, ɳ^2^_p_ = 0.065. In addition, ANCOVA led to a significant interaction of condition x group, *F*(1.81, 488) = 5.97, *p* = 0.004, ɳ^2^_p_ = 0.022. The interaction effect showed that the difference between the two performance groups becomes more pronounced with increasing difficulty. Also, significantly longer durations were observed in conditions with increased cognitive load. An interaction effect condition x age could also be reported *F*(1.81, 488) = 19.1, *p* < 0.001, ɳ^2^_p_ = 0.066. The post-hoc analysis showed that the durations in TDT-B (*M* = 40.8, *SE* = 0.539) were significantly higher than in TDT-A (*M* = 33.3, *SE* = 0.406) or the motor (TDT-M) condition (*M* = 24.7, *SE* = 0.290) (*p* < 0.001). Also, the *post hoc* analysis showed that in the TDT-M, *t*(273) = 2.13, *p* = 0.035, *d* = 0.257, the TDT-A, *t*(270) = 3.44, *p* = 0.001, *d* = 1.55 and the TDT-B, *t*(272) = 5.15, *p* < 0.001, *d* = 1.44, both groups differed significantly from each other, the HP group always outperformed the LP group.

### 3.3 Motor-cognitive interferences in the non-soccer-specific task (TWT) and the soccer-specific task (TDT)

#### 3.3.1 Trail-walking test

**Figure 4:**
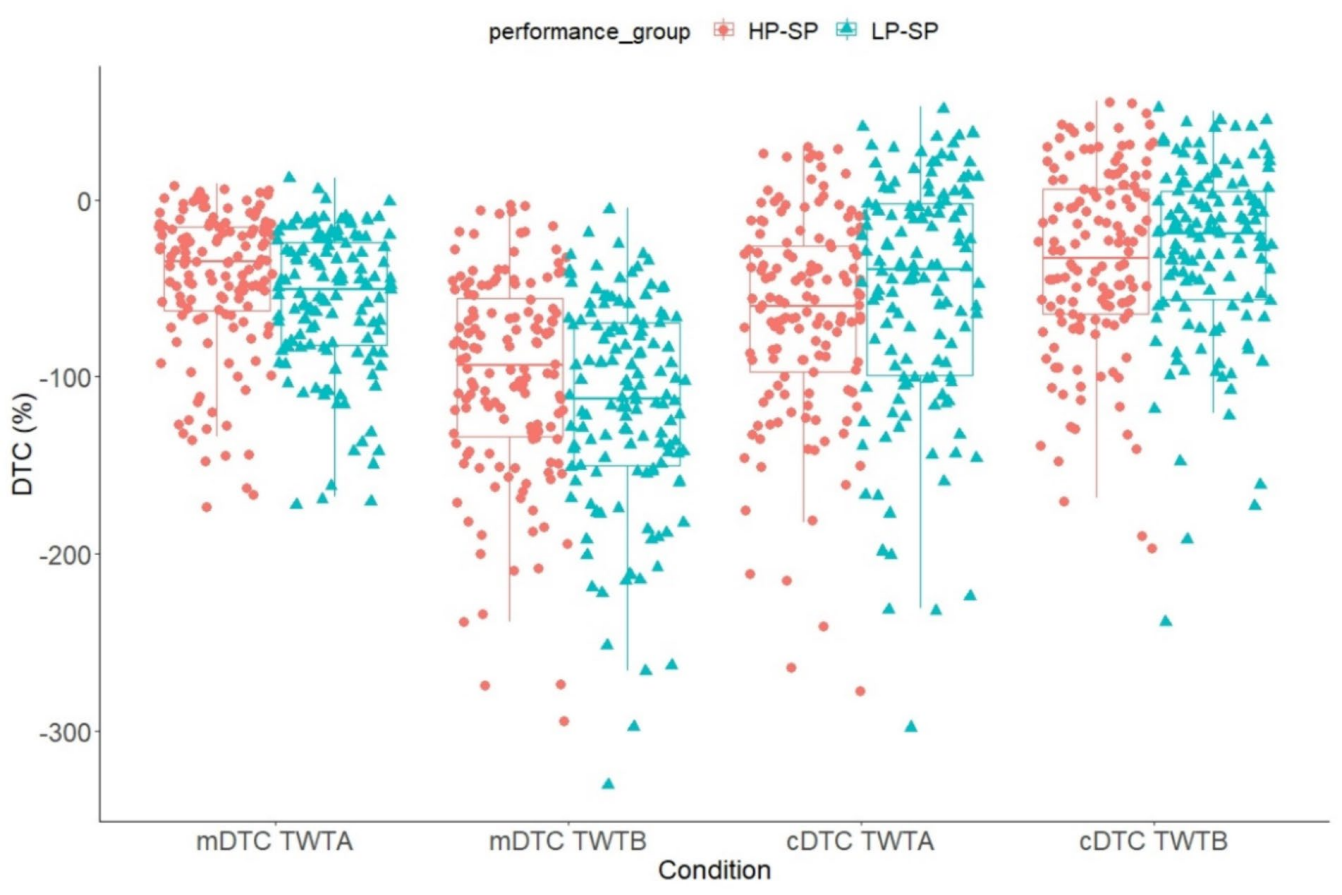
Means and standard deviation of the motor and cognitive DTC in the TWT (TWT-A, TWT-B) divided into high- and low-performance groups. TWT-M, Trail-Walking Test – pure motor task; TWT-A, Trail-Walking Test – numbers; TWT-B, Trail-Walking Test – numbers & letters.

Regarding the proportional DTC in the non-soccer specific task (TWT), a 2 (group: HP-SP vs. LP-SP) x 2 (condition: high vs. low cognitive load) x 2 (domain: cognitive vs. motor) ANCOVA with repeated measurements and age as covariates were calculated. The results showed a significant main effect domain, *F*(1, 270) = 31.9, *p* < 0.001 ɳ^2^_p_ = 0.106, with greater interference for the motor task (motor: *M* = −79.5, *SE* = 2.44; cognitive: *M* = −45.5, *SE* = 2.87) (*p* < 0.001). A significant interaction effect could be observed for domain x age, *F*(1, 270) = 71.5, *p* < 0.001, ɳ^2^_p_ = 0.209, as well as for condition x age, *F*(1, 270) = 4.08, *p* = 0.044, ɳ^2^_p_ = 0.015, and domain x condition, *F*(1, 270) = 67.1, *p* < 0.001, ɳ^2^_p_ = 0.199. Under low cognitive load, comparable motor and cognitive DTC were observed (motor: *M* = −51.1, *SE* = 2.15; cognitive: *M* = −59.2, *SE* = 3.67). With increased cognitive load, motor DTC was higher (motor: *M* = −107, *SE* = 3.34; cognitive: *M* = −31.9, SE = 3.08). The Pearson correlation coefficient for the relationship between age and motor or cognitive DTC showed that motor DTC increases with age (TWT-A: *r* = −506, *p* < 0.001; TWT-B: *r* = −0.384, *p* < 0.001) and cognitive DTC became less apparent with increasing age (TWT-A: *r* = 0.268, *p* < 0.001; TWT-B: *r* = 0.136, *p* < 0.024). A significant group difference in DTC in the non-soccer-specific task could not be observed (see Figure 4).

#### 3.3.2 Trail-dribbling test

**Figure 5:**
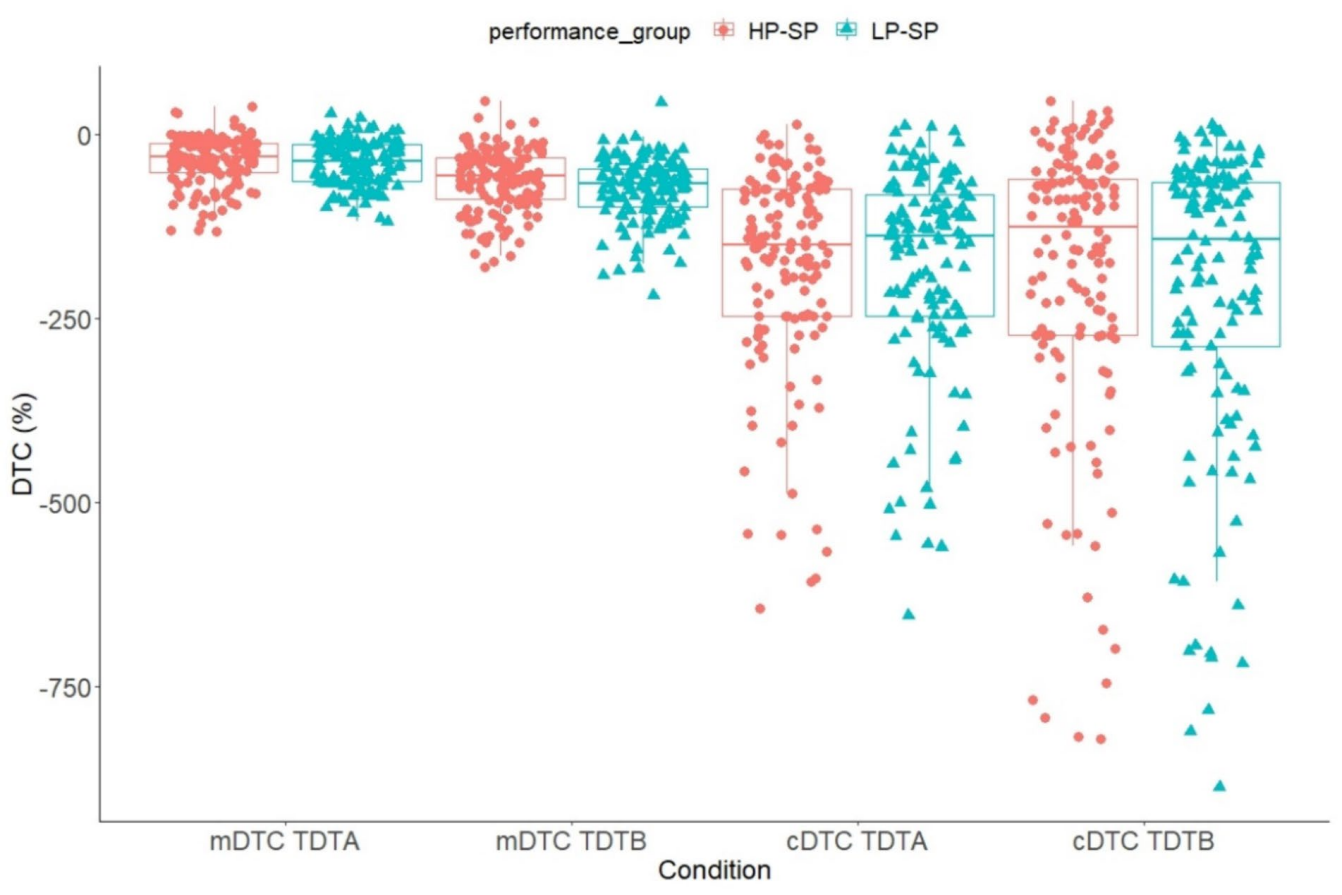
Means and standard deviation of the motor and cognitive DTC in the TDT (TDT-A, TDT-B) divided into the high- and low-performance groups. TDT-M, Trail-Dribbling Test – pure motor task; TDT-A, Trail-Dribbling Test – numbers; TDT-B, Trail-Dribbling Test – numbers & letters.

Regarding the proportional DTC in the soccer-specific task (TDT), a 2 (group: HP-SP vs.- LP-SP) x 2 (load: high vs. low cognitive load) x 2 (domain: cognitive vs. motor) ANCOVA with repeated measurements and age as covariates were calculated. The results showed a significant main effect domain, *F*(1, 270) = 4.35, *p* = 0.038 ɳ^2^_p_ = 0.016, with greater DTCs for the cognitive task (motor: *M* = −53.4, *SE* = 1.87; cognitive: *M* = −190, *SE* = 9.49) (*p* < 0.001). A significant interaction effect could be observed for domain x age, *F*(1, 270) = 4.24, *p* = 0.040, ɳ^2^*_p_* = 0.015, as well as for condition x age, *F*(1, 270) = 17.9, *p* < 0.001, ɳ^2^_p_ = 0.062, and domain x load, *F*(1, 270) = 19.6, *p* < 0.001, ɳ^2^_p_ = 0.068. Both motor (TDT-A: *M* = −38.1, *SE* = 1.74; TDT-B: *M* = −68.7, *SE* = 2.38) and cognitive (TDT-A: *M* = −178, *SE* = 8.23; TDT-B: *M* = −201, *SE* = 11.6) DTCs were greater under increased cognitive load. The Pearson correlation coefficient for the relationship between age and motor or cognitive DTC showed that motor (TDT-A: *r* = −468, *p* < 0.001; TWT-B: *r* = −0.435, *p* < 0.001) and cognitive (TDT-A: *r* = −0.149, *p* = 0.014; TDT-B: *r* = −0.261, *p* < 0.001) DTCs increased with age. A significant difference in the DTC between groups in the soccer-specific task could not be observed (see Figure 5).

**Figure 6:**
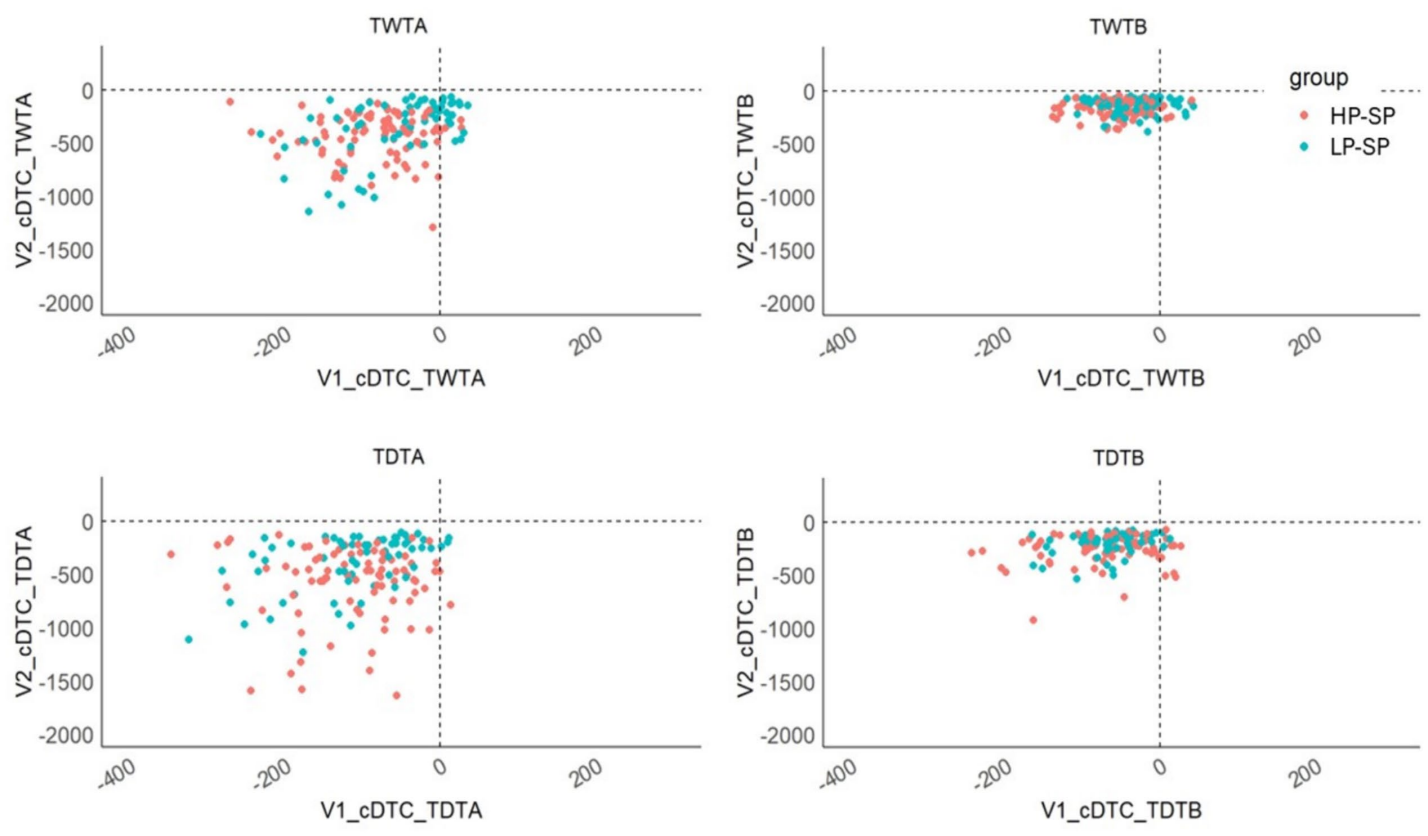
Scatter plot comparing the calculation methods for cognitive DTC under TWT and TDT conditions. V1, Calculation of DTCs based on cognitive performance with the signaling task; V2, Calculation of the DTCs based on cognitive performance with the formula (DT – motor ST); cDTC, cognitive dual-task costs; TWT, Trail-Walking Test; TDT, Trail-Dribbling Test; HP-SP, high performance soccer players; LP-SP, low performance soccer players.

The following correlations emerged between the calculated pure cognitive performances and performances in the pure cognitive signaling tasks: [TWTA-TWTM] to signaling task: *r* = 0.446, *p* < 0.001; [TWTB-TWTM] to signaling task: *r* = 0.367, *p* < 0.001; [TDTA-TDTM] to signaling task: *r* = 0.340, *p* = 0.001; [TDTB-TDTM] to signaling task: *r* = 0.374, *p* = 0.001. Also, in the calculation of the cognitive DTCs, we observe strong correlations between both approaches: cDTC in TWTA: *r* = 0.263, *p* < 0.001; cDTC in TDTA: *r* = 0.181 *p =* 0.015; cDTC in TDTB: *r* = 0.178, *p =* 0.015. We do not see any significant correlation for cDTC in TWTB: *r* = 0.063, *p* < 0.217 (Figure 6).

### 3.4 ROC – analyses for the durations in the TDT

The TDT was beneficial in distinguishing HP-SP from LP-SP in the age groups between 14 and 15 years (AUC = 0.712–0.820) and between 16 and 17 years (AUC = 0.634–0.839) (Table 3).

**Table 3:** Statistics and thresholds of the receiver-operating-characteristic curves for the TDT (velocities in TDT) to differentiate between high- and low-performance players.

	n	Youden index	Sensitivity	Specificity	AUC	Threshold value	*p* value
12–13 years							
TDT-M	26/55	0.320	0.585	0.731	0.659	26.8	**0.022**
TDT-A	26/55	0.206	0.283	0.923	0.590	27.5	0.198
TDT-B	26/55	0.338	0.415	0.923	0.660	33.9	**0.021**
14–15 years							
TDT-M	15/27	0.781	0.929	0.852	**0.712**	23.6	**0.028**
TDT-A	15/27	0.526	0.786	0.741	**0.743**	28.8	**0.011**
TDT-B	15/27	0.677	0.714	0.963	**0.820**	38.5	**0.001**
16–17 years							
TDT-M	9/31	0.649	0.871	0.778	**0.839**	28.3	**0.002**
TDT-A	9/31	0.323	0.323	1.00	0.634	29.5	0.225
TDT-B	9/31	0.631	0.742	0.889	**0.817**	44.1	**0.004**
18–24 years							
TDT-M	31/56	0.388	0.548	0.839	0.657	18.5	**0.016**
TDT-A	31/56	0.140	0.195	0.946	0.513	24.8	0.842
TDT-B	31/56	0.222	0.258	0.964	0.597	27.6	0.136
25–34 years							
TDT-M	15/10	0.367	0.700	0.667	0.687	19.1	0.120
TDT-A	15/10	0.376	0.700	0.667	0.660	31.6	0.183
TDT-B	15/10	0.300	0.900	0.400	0.633	45.7	0.267

Significant results are shown in bold type; n = HP/LP. TDT-M, Trail-Dribbling Test – pure motor task; TDT-A, Trail-Dribbling Test – numbers; TDT-B, Trail-Dribbling Test – numbers & letters.

## 4 Discussion

### 4.1 Behavioral results

The study’s main objective was to introduce a method for quantifying motor-cognitive soccer-specific dribbling tasks and establish a theoretical foundation for this quantification. Specifically, this method falls within the framework of the navigation DT paradigm, where a motor task (locomotion: here dribbling) and a cognitive task (wayfinding) (adapted from the Trail-Walking-Test, Schott, 2015) are combined and integrated into a large-scale spatial ability task.

Soccer, being an “open” sports game, presents unique challenges to players, requiring simultaneous engagement in both motor and cognitive tasks in almost all situations (Smith and Chamberlin, 1992; Gabbett et al., 2011; Scharfen and Memmert, 2019; Ren et al., 2022). Similar demands exist in other sports, where the ability to perform two tasks concurrently is essential for achieving a solid level of performance. However, this requirement becomes problematic when simultaneously processing different tasks, leading to a performance decrement in the single-task condition. For instance, beginners learning dribbling in basketball or soccer need to focus intensely on the ball, preventing them from observing the game (visual search), tracking the movements of their teammates and opponents, and making a decision about where to “go” next (wayfinding). Dribbling becomes increasingly synchronized and automated with training and practice, allowing players to “clear their heads” to process a second task (Carr et al., 2013). However, reliable guidelines on reaching this state of automation are scarce in relevant training manuals, and empirical evidence supporting such recommendations is usually lacking (Blischke and Reiter, 2002). The underlying concept in studies on cognitive-motor dual tasking is that resources are limited (e.g., Kahneman, 1973; Wickens, 2008), and performance tends to suffer when divided between a cognitive and a motor task. Resource competition is particularly evident in individuals who are in the process of acquiring a new motor skill (Schaefer, 2014). On the other hand, there seem to be certain situations where performing two tasks simultaneously can enhance motor and/or cognitive learning (for an overview, see Wollesen et al., 2022). However, the mechanisms underlying this interaction are not yet fully understood, making reliable predictions about the effects of different DT combinations challenging.

A further objective of this study was to compare the processing times of the Trail-Walking Test (TWT) and Trail-Dribbling Test (TDT) between high-performance soccer players (HP-SP) and low-performance soccer players (LP-SP) and to identify any performance advantages of HP-SP. In this respect, our first hypothesis can be confirmed, indicating that depending on the specificity of the task and the level of cognitive demand, interference effects, and group differences become more pronounced. It is worth noting that in both tasks (TWT and TDT) and across both performance groups, the processing durations increase as the cognitive load intensifies, consistent with numerous studies utilizing the DT paradigm in various domains (Schaefer, 2014). In the TWT, specifically the motor tracking task (TWT-M) without soccer-specific dribbling, there is only a small difference between HP-SP and LP-SP. The superior agility and motor speed of the HP-SP are among the factors that may explain this observation. In contrast, in the TDT, differences between the performance groups are evident in all conditions, with the disparities becoming more pronounced as the additional cognitive load increases. This finding aligns with the studies conducted by Smith and Chamberlin (1992), which also revealed more significant group differences between experts and novices under increased cognitive load. Similar patterns of larger differences in DT compared to ST conditions between experts and novices were observed in the study by Beilock et al. (2002).

Interferences predominantly occur in the cognitive domain, particularly under high cognitive load. Against our expectations, we found no differences in motor or cognitive performance declines between the HP-SP and LP-SP groups. In the TWT test, motor performance declines were greater than cognitive ones in both groups. However, in the TDT test, cognitive performance declines were higher than motor ones in all groups, no matter how difficult the additional task was. This observation is explained by the ability to focus on the cognitive task in the TWT while doing the more straightforward running task (without soccer-specific dribbling). Thus, both groups directed attentional resources toward the cognitive task, requiring only minimal cognitive resources for the running component. In the TDT, however, attention is primarily focused on the motor task, leading to neglect of the cognitive task. Presumably, players in both groups strongly prioritize attention toward the dribbled ball, which hampers visual search for numbers, numbers, and letters, resulting in higher DTC primarily in the cognitive task. On the other hand, this implies that participants have to update their next move constantly. Experts compared to lower-level soccer players are expected to flexibly switch and combine allocentric spatial processing skills [object-based or third-person perspective (relative to the environment)] and egocentric spatial processing, a navigational strategy based on a first-person perspective (relative to the body) because ball games with a high training and competition volume involve the use of this process more than in everyday life (Ekstrom et al., 2003). If they struggle to control the ball and follow a ball that goes the wrong way, they are more likely to “lose track” of the sequence of numbers and letters. In this sense, a lack of soccer-specific dribbling skills makes the ‘in-built’ cognitive task (wayfinding task) more challenging.

The second and third objectives were to evaluate the diagnostic quality of the Trail-Dribbling Test (TDT). Both performance groups were further divided into different age groups to achieve this, allowing for a more detailed assessment of the TDT’s ability to differentiate between HP-SP and LP-SP. Our hypothesis can be confirmed, indicating that with increased cognitive demand, differentiation between HP-SP and LP-SP, especially in soccer-specific motor tasks, becomes evident. Between the ages of 14 and 15, the study demonstrated moderate (TWT-M: AUC = 0.712; TDT-A: AUC = 0.743) and good (TDT-B: AUC = 0.820) diagnostic quality in distinguishing between HP-SP and LP-SP. Moreover, as the additional cognitive load increased, the diagnostic quality of the TDT improved (TDT-M: AUC = 0.712, *p* = 0.013; TDT-A: AUC = 0.743, *p* = 0.008; TDT-B: AUC = 0.820, *p* = 0.001), with the TWT-B showing particularly good sensitivity (71.4%) and specificity (96.3%). Based on these results, it is appropriate to differentiate between the groups, especially using the TDT with high cognitive load (TWT-B), with a threshold value of 38.5 s for processing time. The diagnostic quality ranges from weak (TDT-A: AUC = 0.634) to good (TDT-M: AUC = 0.839; TDT-B: AUC = 0.817) between the ages of 16 and 17. Moderate cognitive load (TDT-M) only allows weak discrimination between HP-SP and LP-SP. Differentiation is most effective in motor soccer-specific dribbling tasks (TDT-M) and DTs with high cognitive load (TDT-B). This can be explained by the fact that for LP-SP, an additional simple cognitive task (TDT-A) leads to lower motor DTCs, possibly due to the automated and self-organized execution of the dribbling task. However, when cognitive load increases beyond a certain point, available resources become insufficient, resulting in significant impairments in LP-SP. This observation is consistent with the findings of Gabbett et al. (2011), who investigated the performance of skilled and lesser-skilled rugby players in a rugby drill under ST and DT conditions while simultaneously performing a verbal tone recognition task. The performance of experts was more resistant to skill decrement under DT conditions. As cognitive demands increase, competition for limited attention resources (Kahneman, 1973; Wickens, 2008) can have a negative impact that outweighs the benefits of an external focus of attention, ultimately leading to decreased performance (Constrained Action Hypothesis; Wulf et al., 2001). In motor tasks, directing attention to the task at hand may result in a performance loss, particularly for LP-SP. Poor diagnostic quality was observed in all other age groups (12–13; 18–24; 25–34 years) with AUC values ranging from 0.590 to 0.687. These results are consistent with the findings of Ljach and Witkowski (2010), who examined the development of coordination skills in 11- to 19-year-old soccer players (*n* = 600). They found that the period from 11 to 13 years was most conducive to the development of coordinative skills, followed by the period from 14 years onwards. This may explain why the discriminatory strength of the TDT is not observed until the age of 14 and may explain why there is no significant discrimination in the years between 12. The reliability of these measurements might have been improved by evaluating multiple trials for each condition. Additionally, the variation in participant numbers across different age groups may have influenced the statistical power of the results. Also, the threshold value for distinguishing performance groups was determined using the Youden Index. Alternatively, one could determine the desired sensitivity and evaluate the test’s specificity. If the objective is to identify as many high-performing individuals as possible, a predetermined sensitivity value should be utilized. The test’s specificity, which refers to its ability to identify low-performing individuals, can be considered secondary to the primary goal of talent identification. If a high percentage of players are falsely identified as test positive (false-positive) at low specificity, it may not have severe consequences as late-developing athletes may not be prematurely excluded.

### 4.2 Methodological suggestions

As Ali (2011) pointed out, research and practice often neglect the cognitive component when assessing skills and identifying talent. The cognitive component involves decision-making and information processing, which are a fundamental part of skill development. A motor-cognitive DT approach has been proposed to address this, aiming to integrate both domains. The DT method falls under the “Expert Performance Approach” (Mann et al., 2007) as it involves testing athletes under ecologically valid conditions using tasks such as visual search and cognitive flexibility that are representative of open sports like soccer. DT approaches allow for assessing skill automation (in this case, dribbling) and evaluating specific training methods to automate skills. Automation of skills is crucial since many skills practiced in training can break down under pressure or additional cognitive demands during real-game situations.

McIsaac et al. (2015) created a framework for a DT taxonomy to guide the discussion of existing evidence-based studies on interferences in DTs within a broader framework. This enables summative statements and leads to clarity and a better understanding of the research area. According to this classification scheme, tasks such as ‘carrying a cup while walking’ or ‘talking on a mobile phone while walking’ are not considered dual tasks (DTs). However, our perspective differs somewhat. We contend that in these scenarios, it is possible to entirely redirect our attentional resources from one activity (such as talking) to another (such as walking) despite the tasks not being entirely independent of each other. Therefore, we can also refer to a DT paradigm in our approach. McIsaac et al. (2015) classified tasks using a two-dimensional system that assesses ‘Novelty’ (familiarity with the task) and ‘Task Complexity’ (attention required, number of components, and degrees of freedom involved), ranging from ‘High’ to ‘Low.’ The level of attentional demand imposed by a secondary task may vary depending on the individual. For example, tasks that require mathematical skills may only require minimal mental effort if the individual is well-practiced in them. The same applies to soccer-specific experts and novices when it comes to soccer-specific motor tasks (TDT). As learning progresses, tasks can become more challenging by reducing the playing field, adding more numbers and letters, starting with different numbers with varying distances (e.g., 3–24-678) or different letters (e.g., C-E-G), or introducing a third feature (e.g., opponent figures with increasing size). Our approach aligns with McIsaac’s taxonomy (refer to Table 4).

**Table 4:** Exemplary integration of the TDT into the dual-task framework by McIsaac et al. (2015) for HP-SP (experts) and LP-SP (novices).

Group	Type of task	Task novelty	Task complexity	
HP-SP			Low	High
	Dual cognitive-motor	Low	TDT-A	TDT-B
		High		
Group	Type of task	Task novelty	Task complexity	
LP-SP			Low	High
	Dual cognitive-motor	Low		
		High	TDT-A	TDT-B

HP-SP, high-performance soccer players; LP-SP, low-performance soccer players; TDT-A, Trail-Dribbling Test with numbers; TDT-B, Trail-Dribbling Test with numbers & letters.

An alternative method for operationalizing cognitive performance in the TWT and TDT involves having the players stand in the center of the field and use a laser pointer to indicate numbers and letters (signaling task). This behavior is reminiscent of actions in soccer, where players signal running paths or provide specific positional instructions to their teammates. Simultaneously, this approach enhances the external validity of our procedure. Through this specific implementation of the cognitive task within the context of the TWT and TDT, ST cognitive performance can also be assessed. We applied this approach to a subset of our sample (*n* = 165) in addition to the aforementioned methods, and we identified strong correlations when comparing it to the calculated ST cognitive performance. This emphasizes the validity of operationalizing cognitive performance through signaling tasks or the calculation method used in our study as an effective approach for assessing cognitive performance in single tasks (STs) and calculating cognitive DTC.

Even though we did not conduct a longitudinal study that can confirm an effect over time, the TDT could potentially be used as a training tool to automate skills such as dribbling by incorporating an additional cognitive task. The difficulty level can be adjusted based on performance level by modifying the task, such as increasing the number of letters, starting at different numbers or letters (e.g., D-13-E-14), or extending the playing field. Variations such as dribbling the numbers and letters backward or dribbling letters corresponding to predefined terms/names are also possible variations. These variations aim to maintain high cognitive demands since the cognitive task of connecting numbers and letters in ascending order becomes easier or automated over time. Randomized changes in the stimuli can be introduced to maintain the visual search demands and enhance spatial orientation. These variations offer more motivation compared to the monotonous execution of the described TDT. Additionally, expanding the playing field accentuates different types of dribbling, such as “space-gaining” dribbling, requiring more considerable distances, particularly in larger playing fields. Conversely, smaller playing fields can emphasize “ball-keeping” dribbling. The selection of adjustments depends on specific requirements and training objectives, allowing for flexibility in the training approach.

## 5 Conclusion, limitations, and future directions

The study’s results offer a new perspective on navigation research in ball sports, specifically soccer. They demonstrate that spatial disorientation, along with limitations in dribbling performance, can be effectively induced and evaluated in adolescent soccer players through the Trail-Dribbling Test. This is evidenced by the significantly higher dual-task costs observed in the high cognitive load condition.

Therefore, the TDT could be a suitable screening and training instrument for individuals aged 14 to 17 years. It is capable of observing more significant differences between performance groups as cognitive load increases. However, it is important to note that the TDT has not been compared to any gold-standard screening tools. Furthermore, the usefulness of this approach may be restricted due to its narrow age range (based on the results of this study). In soccer, talent identification and selection often depend on subjective evaluations made by experienced coaches (Christensen, 2009). Most studies in this field focus primarily on physiological aspects (e.g., endurance and speed) and anthropometric characteristics (i.e., height and weight) of players (Murr et al., 2018). However, these approaches may inadvertently exclude late-maturing individuals. The authors recommend considering multiple physiological measures in addition to the mentioned anthropometric characteristics.

However, using only a few test procedures to make selection decisions for the future success of young athletes is highly unsatisfactory. A holistic approach to talent identification should encompass physical resources, physiological characteristics, motivational factors, social opportunities, family support, personality traits, and cognitive-volitional features of athletes. Unfortunately, many of these talent criteria often go unconsidered. Therefore, it is not recommended to create or support selection criteria or make probability statements about future success based solely on one test. Additionally, the term ‘talent’ has multiple interpretations, and there are no universally accepted criteria to define the concept. This highlights the existence of various approaches to examining talent. To develop athletes’ talent to its fullest potential, researchers and coaches should prioritize maximizing the factors that contribute to their success. The TDT assesses both cognitive and motor skills, making it a valuable tool in this regard.

When categorizing athletes as high-performing or low-performing, it is important to note that athletes competing in the “Landesliga” may not necessarily be considered ‘experts’. This distinction could potentially impact the interpretation of the results. While the study aimed to include participants with varying levels of performance, it is crucial to recognize that the definition of ‘expertise’ can vary depending on the context and sport being studied. In this context, including Landesliga athletes in a soccer-specific study could potentially bias the results, particularly if they are perceived as less experienced or competent than athletes at higher performance levels (Bundesliga). Future research should carefully consider participant selection and competence definition to avoid such biases. It is crucial to consider other relevant factors, such as experience, training intensity, or individual performance history when assessing performance, rather than relying solely on the competition level. Categorizing participants into high-performance (HP) and low-performance (LP) groups by calculating a score could provide a more detailed and precise classification. Future research could investigate the feasibility of integrating both league classification and a calculated score based on relevant performance variables. This integration could lead to a more comprehensive and nuanced understanding of participants’ performance levels, increasing the validity and depth of the results. This strategy requires careful consideration of the weighting and selection of performance variables to ensure that the derived scores accurately reflect athletes’ abilities while remaining practical and interpretable.

Moving forward, a more comprehensive approach to talent identification and development in sports is necessary. Researchers and coaches should focus on maximizing athletes’ potential by optimizing various factors, including cognitive and motor aspects. Further integration of non-traditional talent criteria, such as motivational factors, social support, and personality traits, could help create a more holistic picture of talents. Future research approaches could involve refining and adapting motor-cognitive decision-making tasks. By utilizing different variations and tasks in the decision-making process, athletes’ flexibility and adaptability could be further enhanced. Integrating modern technologies, such as virtual reality, could offer new opportunities for training and assessing cognitive and motor skills. Future studies and practices in talent identification and development in sports should encompass a broader range of factors and criteria to gain a more comprehensive understanding of talents and ensure that no potential goes unnoticed. This approach can facilitate the optimal development and nurturing of athletes to help them achieve their full potential.

## Data availability statement

The raw data supporting the conclusions of this article will be made available by the authors, without undue reservation.

## Ethics statement

The studies involving humans were approved by Commission for Responsibility in Research (Ethics Committee) of the University of Stuttgart. The studies were conducted in accordance with the local legislation and institutional requirements. Written informed consent for participation in this study was provided by the participants’ legal guardians/next of kin.

## Author contributions

TJK: Writing – review & editing, Writing – original draft, Visualization, Validation, Methodology, Investigation, Formal analysis, Data curation, Conceptualization. NS: Writing – review & editing, Writing – original draft, Visualization, Validation, Methodology, Investigation, Formal analysis, Data curation, Conceptualization.
